# Engineered hyaluronic acid-decorated niosomal nanoparticles for controlled and targeted delivery of epirubicin to treat breast cancer

**DOI:** 10.1016/j.mtbio.2022.100349

**Published:** 2022-07-06

**Authors:** Amirreza Mansoori-Kermani, Sadaf Khalighi, Iman Akbarzadeh, Fazeleh Ranjbar Niavol, Hamidreza Motasadizadeh, Athar Mahdieh, Vahid Jahed, Masoud Abdinezhad, Nikoo Rahbariasr, Mahshid Hosseini, Nima Ahmadkhani, Behnam Panahi, Yousef Fatahi, Masoud Mozafari, Alan Prem Kumar, Ebrahim Mostafavi

**Affiliations:** a Department of Chemical and Petrochemical Engineering, Sharif University of Technology, Tehran, Iran; b Department of Stem Cells and Developmental Biology, Cell Science Research Center, Royan Institute for Stem Cell Biology and Technology, ACECR, Tehran, Iran; c Department of Pharmaceutical Nanotechnology, Faculty of Pharmacy, Tehran University of Medical Sciences, Tehran, Iran; d Rudolfs Cimdins Riga Biomaterials Innovations and Development Centre of RTU, Institute of General Chemical Engineering, Faculty of Materials Science and Applied Chemistry, Riga Technical University, Pulka St. 3/3, Riga, LV, 1007, Latvia; e School of Chemical Engineering, College of Engineering, University of Tehran, Tehran, Iran; f Polymer Research Laboratory, Department of Chemistry, Sharif University of Technology, Tehran, Iran; g Nanotechnology Research Centre, Faculty of Pharmacy, Tehran University of Medical Sciences, Tehran, Iran; h Department of Tissue Engineering and Regenerative Medicine, Faculty of Advanced Technologies in Medicine, Iran University of Medical Sciences, Tehran, Iran; i Department of Pharmacology, Yong Loo Lin School of Medicine, National University of Singapore, Singapore 117600, Singapore; j NUS Center for Cancer Research, Yong Loo Lin School of Medicine, National University of Singapore, Singapore 117597, Singapore; k Stanford Cardiovascular Institute, Stanford University School of Medicine, Stanford, CA, 94305, USA; l Department of Medicine, Stanford University School of Medicine, Stanford, CA, 94305, USA

**Keywords:** Nanomedicine, CD44, Active targeting, Breast cancer, Hyaluronic acid, Controlled drug delivery

## Abstract

Targeted drug delivery systems using nanocarriers offer a versatile platform for breast cancer treatment; however, a robust, CD44-targeted niosomal formulation has not been developed and deeply studied (both *in vitro* and *in vivo*) yet. Here, an optimized system of epirubicin (Epi)-loaded niosomal nanoparticles (Nio) coated with hyaluronic acid (HA) has been engineered for targeting breast cancer cells. The nanoformulation was first optimized (based on size, polydispersity index, and entrapment efficiency); then, we characterized the morphology, stability, and release behavior of the nanoparticles. Epirubicin release from the HA-coated system (Epi-Nio-HA) showed a 21% (acidic buffer) and 20% (neutral buffer) reduction in comparison with the non-coated group (Epi-Nio). The cytotoxicity and apoptosis results of 4T1 and SkBr3 cells showed an approximately 2-fold increase in the Epi-Nio-HA system over Epi-Nio and free epirubicin, which confirms the superiority of the engineered nanocarriers. Moreover, real-time PCR data demonstrated the down-regulation of the MMP-2, MMP-9, cyclin D, and cyclin E genes expression while caspase-3 and caspase-9 gene expression were up-regulated. Confocal microscopy and flow cytometry studies uncovered the cellular uptake mechanism of the Epi-Nio-HA system, which was CD44-mediated. Furthermore, *in vivo* studies indicated Epi-Nio-HA decreased mice breast tumor volume by 28% (compared to epirubicin) without side effects on the liver and kidney. Conclusively, our results indicated that the HA-functionalized niosomes provide a promising nanoplatform for efficient and targeted delivery of epirubicin to potentially treat breast cancer.

## Graphical abstract

After optimizing the formulation of epirubicin-loaded niosomes, the optimized nanoparticles were coated with hyaluronic acid to provide active targeting and more efficient delivery.

**Figure:**
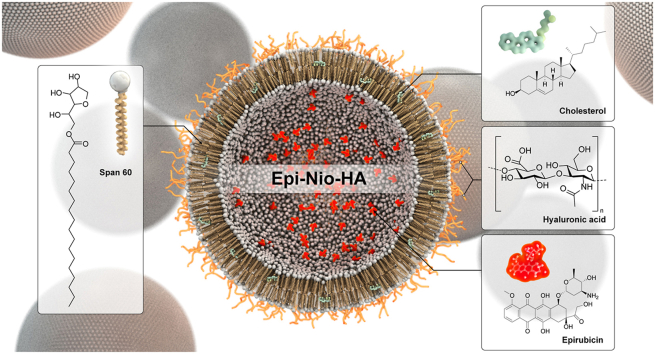

## 1 Introduction

Cancer is a serious, global health issue and the second main death cause in the United States [1]. In 2018, breast cancer was identified in 12% of women in the United States during their lifetimes [2]. Chemotherapy, as one of the inseparable parts of cancer treatments, involves toxicities, namely asthenia, edema, myalgias, myelosuppression (anemia, neutropenia, thrombocytopenia), and sensory neuropathy [3]. Herein, the focus is on the epirubicin drug, which is one of the chemotherapeutic drugs; anthracycline derivative of doxorubicin with a similar mechanism of action and comparable antitumor activity [4]. Epirubicin has a more biocompatible therapeutic index than doxorubicin, and it has undergone significant assessment in the treatment of breast cancer [5,6]. Extravasation of epirubicin into subcutaneous tissues can cause irritation and may lead to major local damages, including ulceration. Therefore, there are efforts for adverse effects elimination with different nanocarriers for the drug due to the current challenges of using chemotherapeutic medicines in the clinic [7,8]. Moreover, vesicular drug delivery has been introduced to reduce the treatment cost by increasing the drug bioavailability and also represented solutions for drug instability and insolubility issues [9].

Among different vesicles in nanomedicine, niosomes, which are made of non-ionic surfactants, have shown a great potential to be used as drug carriers [[10], [11]]. In comparison with other vesicles like liposomes, niosomes possess improved bioavailability, enhanced stability, higher entrapment efficiency (EE), low toxicity that comes from their no-ionic nature, and they are easy to prepare at a lower cost [12,13]. Although nano-sized niosomes can be gathered in tumor tissues, this is a passive targeting since a considerable amount of the drug will be released before niosomes cell translocation via receptor-mediated endocytosis [14]. Thus, targeting of receptors available on the cell surface has recently emerged as an advanced drug delivery strategy to overcome the problems related to passive targeting [15,16].

The cluster-determinant 44 (CD44) is a cell-surface glycoprotein, which is overexpressed on tumor cells’ surfaces, namely lung cancer, pancreatic cancer, and breast cancer tumors [17,18]. *In vivo* evidence proved that CD44 induces breast cancer metastasis to the liver [19]. Hence, designing anticancer drug delivery systems aiming at CD44 receptor targeting would be a successful strategy. In this regard, Hyaluronic Acid (HA), which is a natural polysaccharide and also one of the main constituents of the extracellular matrix (ECM), has been widely studied in previous literature [16,[20], [21]]. This is mainly due to its biocompatibility, biodegradability, the potential to enhance bioavailability and reduce toxicity, as well as the fact that it does not have synthetic polymers drawbacks [16,22,23]. More interestingly, HA specifically binds to CD44 and improves intracellular anticancer drug delivery to various cancerous cells, including breast cancer cells; therefore, HA-containing systems can act as favorable anticancer drug carriers for effective chemotherapy [14,18,22,[24], [25], [26], [27]].

**Fig. 1:**
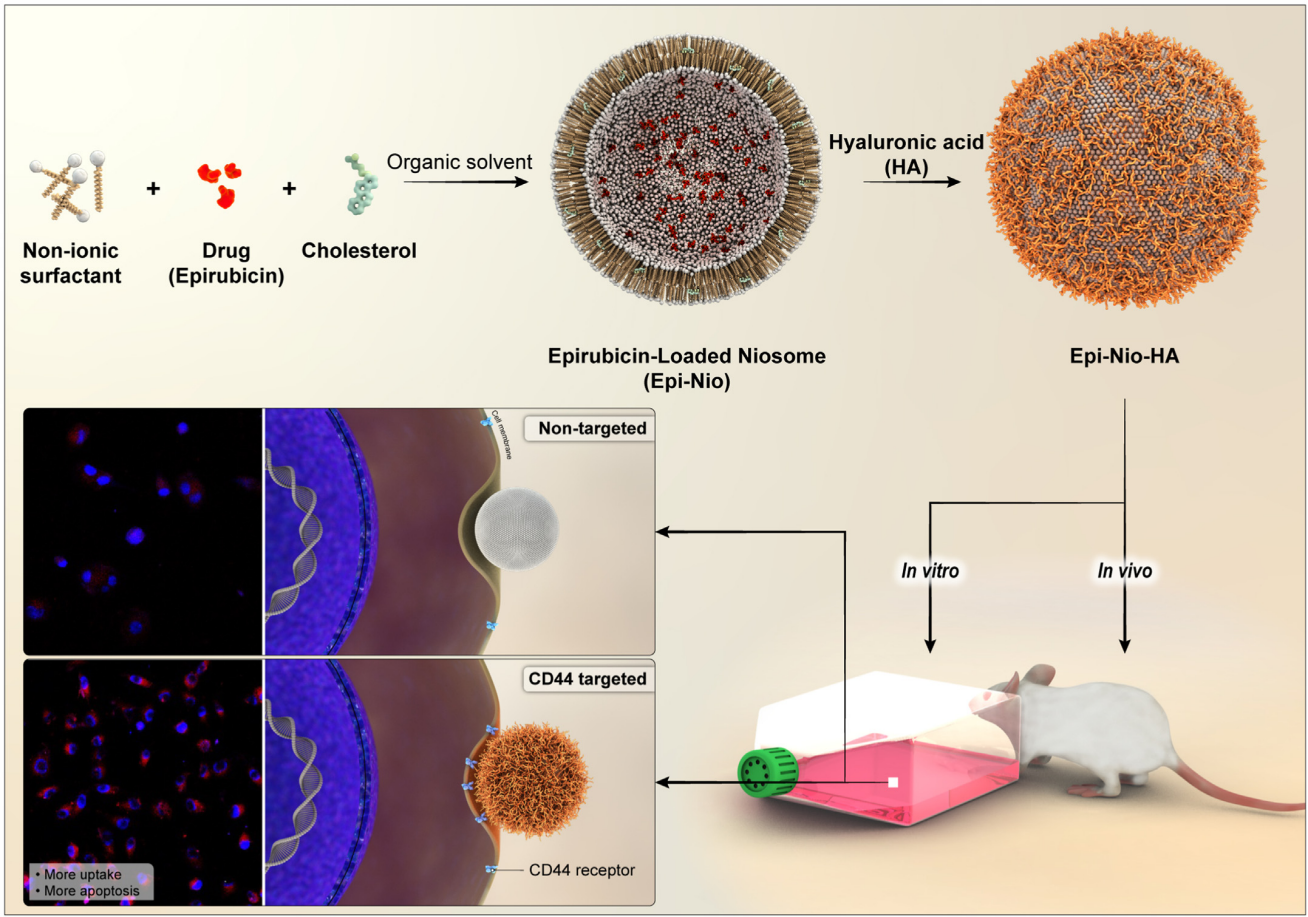
Schematic representation of the structure of Epi-Nio and Epi-Nio-HA nanoparticles, biological experiments, and CD44 receptor-mediated endocytosis.

In this study, we hypothesize that niosomes in combination with HA coating provide a suitable drug carrier for active targeting delivery of epirubicin and eventually breast cancer treatment. This structure seemed to support not only releasing epirubicin in target tumor tissue (passive targeting), but also enhanced cellular uptake (active targeting), in comparison with only epirubicin and Epi-Nio. The primary goal of the design was to enable the delivery system to accumulate in tumor tissue; therefore, we employed pH-sensitive materials (niosome). Taking one step further, we aimed to develop a targeted drug delivery system to improve the internalization of epirubicin into the cancer cells (active targeting). Hence, we conjugated the niosomes with hyaluronic acid hydrogel. First, we optimized the epirubicin-niosomes system in terms of size, EE, release rate, and stability. Second, combining niosomes with HA, we synthesized the final group. Using the same characterization methods in the optimization process, we assessed different groups’ properties as engineered drug carriers compared to epirubicin alone. Moreover, we have conducted cellular experiments to evaluate Epi-Nio-HA and Epi-Nio ability to combat two different breast cancer cells. In addition, the cellular uptake mechanism of the nanoparticles has been scrutinized by confocal microscopy and flow cytometry. Finally, we have carried out animal studies on mice bearing breast cancer tumors to assess the Epi-Nio-HA and Epi-Nio potential for tumor suppression. Fig. 1 shows the followed procedure schematically.

## 2 Materials and methods

### 2.1 Materials

Methanol, ethanol, chloroform, Cholesterol, DMSO, Amicon (Ultra-15-Membrane, MWCO 30000 ​Da), Span 20, Span 60, and Span 80 have been provided from Merck, Germany. Hyaluronic acid (MW 158 ​KDa) was taken from Changzhou Institute of Matera Medica Co. LTD. Dulbecco's Modified Eagle's medium (DMEM), fetal bovine serum (FBS), and penicillin-streptomycin solution were provided from PAN Biotech (Aidenbach, Germany). Trypsin-EDTA, Trypan blue, RPMI-1640 *medium*, PBS and 3-(4,5-dimethylthiazol-2-yl)-2,5-diphenyltetrazolium Bromide (MTT) were bought from Gibco, USA. Dialysis membrane (MWCO 12000 ​Da), Nile red, and Coumarin 6 were obtained from Sigma, USA. Human breast cancer cell lines, MCF- 7 and MDA-MB-231, NIH-3T3, SkBr3, and 4T1 cell lines were obtained from Pasteur Cell Bank, Iran. Epirubicin was purchased from Sina Darou, Iran. Annexin V/propidium iodide (PI) assay (i.e., Dead cell apoptosis kit) was purchased from Roche, Germany. RNA extraction kit Nucleic acid extraction kit was received from Qiagen, United States. The cDNA synthesis was done with Revert AidTM First Strand cDNA Synthesis Kit (Fermentas, Vilnius, Lithuania).

### 2.2 Optimization

To reach the optimum formulation, in which the smaller size, minimum polydispersity index (PDI), and maximum EE are satisfied, two independent variables (i.e., surfactant: cholesterol molar ratio, and drug concentration) were examined. In order to evaluate these factors, the Box–Behnken design through Design-Expert 10.0.3 software (Stat-Ease Inc., Minnesota, U.S.A) was applied. Tables S1 and S2 represent the levels of independent variables. Besides, the percentage error between the observed and expected data was calculated. Eventually, the optimum formulation was selected for the next steps.

### 2.3 Preparation of epirubicin-loaded niosome (Epi-Nio)

Thin-film layer method was applied to load epirubicin in niosomes. In brief, a specified amount of surfactants and cholesterol were dissolved in an organic solvent (chloroform and methanol with a 2:1 ratio). After that, the solvent was evaporated using a rotary vacuum (Heidolph Instruments, Schwabach, Germany) at 60 ​°C and 150 ​rpm until the dried thin layer was formed. Afterward, the dry thin films were hydrated using a drug solution (1 ​mg/mL epirubicin in PBS for 30 ​min (150 ​rpm, 25 ​°C). Eventually, to reach the uniform size distribution, the prepared samples were sonicated for 5 ​min (UP50H compact laboratory homogenizer, Hielscher Ultrasonic, Teltow, Germany). The samples were stored in a refrigerator (at 4 ​°C) for the following tests.

### 2.4 Preparation and coating of HA on Epi-loaded niosomes

For coating hyaluronic acid on niosomal nanoparticles, 10 ​mL of 0.1% (w/v) HA solution was poured dropwise to the epirubicin-loaded niosomes with simultaneously stirring for 1 ​h at room temperature.

### 2.5 Characterization

#### 2.5.1 Fourier-transform infrared spectroscopy (FTIR)

Lyophilized samples of Epi-Nio-HA and all the other elements of drug-loaded niosomes were mixed with KBr in the form of pellet by a hydraulic strain and then analyzed using PerkinElmer FTIR spectrophotometer (spectrum Two, USA). The procedure was performed in the scanning range of 4000–400 ​cm^−1^ in a constant resolution of 4 ​cm^−1^.

#### 2.5.2 Entrapment efficiency

To measure EE, ultra-filtration was employed using Amicon Ultra-15-membrane (MWCO 30 ​kDa) at 4000×*g* for 30 ​min. In summary, after pouring a quantified amount of samples on the top chamber, the amount of non-loaded epirubicin passes through the membrane. The concentration of non-loaded epirubicin was determined using UV–visible spectroscopy (JASCO, V-530, Tokyo, Japan) at maximum absorbance of the drug molecule wavelength (480 ​nm). Eventually, the below equation has been used to calculate the EE percentage.

(1) Entrapment ​Efficiency ​(%) ​= ​[(X ​- ​Y)/X] ​× ​100

where X is the initial drug entrapped into the nanoparticles, and Y refers to the amount of the drug permeated across the membrane.

#### 2.5.3 PDI, size, and morphology

Generally, before conducting any biological experiment, the evaluation of physicochemical properties must be done. In this study, size and PDI were determined by the DLS technique. Briefly, to inhibit the multi-scattering phenomenon, the samples were diluted in double-distilled water in a proportion of 1:100. Then, the experiment was pursued by a 45 ​mm focus lens and a beam length of 2.4 ​mm (Malvern Instrument Ltd., Malvern, UK) at a temperature of 25 ​°C. The morphology and shape of niosomes were also assessed using transmission electron microscopy (TEM) (H-600, Hitachi, Japan, imaged at 100 ​kV) and field emission scanning electron microscopes (FESEM) (Tescan, MIRA3, at an accelerating voltage of 15 ​kV). The triplicate measurements for all samples were carried out, and the data were reported as mean ​± ​SD.

#### *2.5.4* *In vitro* drug release study and kinetic modeling

For *in vitro* drug release study, different forms of drug formulation were analyzed using a dialysis bag membrane. Briefly, explaining the procedure, the dialysis bag containing 2 ​mL of each sample was continuously stirred (50 ​rpm) at 37 ​°C in the PBS solution (50 ​mL, T ​= ​37 ​± ​1 pH ​= ​5.4, 7.4). To calculate the concentration of released drug in PBS solution, at different time intervals, a specified amount of aliquots was taken and read by spectrophotometer method and then replaced by the same amount of fresh PBS solution. The drug release test has been repeated for three times.

To obtain the drug release profile and mechanism, linear kinetic models were applied to the release data. The usual models used for linear forms are as follows:

(a) Zero-order model (cumulative drug released % vs. time)
(b) First-order model (log (drug retained %) vs. time)
(c) Higuchi model (cumulative drug released % vs. square root of time)

(d) Korsmeyer–Peppas model (log (amount of drug released) vs. log (time))

#### 2.5.5 Stability studies

To assess the long-term stability of niosomal formulation, three factors (i.e. size, PDI, and EE) of identical samples at two different temperatures of 4 ​°C and 25 ​°C and different time intervals were measured.

### 2.6 Cytotoxicity

The cytotoxicity of the nanoparticles was performed on two types of breast cancer cell lines: 4T1 (mouse mammary carcinoma) and SkBr3 (human breast cancer cell line) cells MTT assay. Briefly, the cells were maintained in 96-well plates with 10^4^ ​cells per well in a medium containing 1% penicillin-streptomycin and 10% FBS, in an incubator (at 37 ​°C and 5% CO_2_ concentration) for 48/72 ​h. The Epi, Epi-Nio, and Epi-Nio-HA were administrated into the seeded cells. After 48 ​h of incubation, the medium of each well was changed by 20 ​μL of MTT solution (5 ​mg/mL) and incubated for 4 ​h (T ​= ​37 ​°C in a 5% CO_2_ atmosphere). The resulting formazan produced by the living cells was dissolved by 100 ​μL of isopropanol, and absorbance was observed at 570 ​nm using an ELISA Reader (Organon Teknika, Oss, Netherlands). Subsequently, the cytotoxicity rate was calculated by a comparison between the treated cells' absorbance and the untreated cells’ results as a control. Each experiment was performed three times.

### 2.7 Annexin V-FITC vs propidium iodide (PI) assay

4T1 and SkBr3 cells (1 ​× ​10^5^ ​cells/well) were treated with samples (IC50 concentration) for 48 ​h. The cells were washed two times with a cold sterile PBS (pH 7.4) and resuspended in 250 ​mL binding buffer provided by the Apoptosis detection kit. Then the cell death was examined using Annexin V/PI assay, based on the manufacturer's instructions. Eventually, the cell suspensions were transferred to a flow cytometric tube, and the levels of necrotic/apoptotic cells were measured by flow cytometry (BD Biosciences, Singapore).

### 2.8 Gene expression, real-time PCR

For total RNA extraction and cDNA synthesis, respectively, Qiagen (Germantown, MD) and Revert Aid™ First Strand cDNA Synthesis Kit (Fermentas, Vilnius, Lithuania) were used according to the manufacturers' instructions. The data in Table S3 represent the primers’ sequence of the specific genes, caspase-3 and 9, MMP-2 and 9, Cyclin D and E. Eventually, the real-time PCR reaction was carried out using a Light cycler (Bioneer, Daejeon, South Korea) at a temperature program. The relative gene expression was estimated using the ΔΔCt technique, which is described in the literature, presuming that PCR is 100% efficient [28].

### 2.9 Cellular uptake

Confocal microscopy and flow cytometry analyses were performed to investigate the precise localization and cellular uptake of nanoparticles inside the cells [29]. For this purpose, two types of CD44-positive breast cancer cell lines (MDA-MB-231 and MCF-7) and one CD44-negative fibroblast cell line (NIH-3T3) were used. The inhibition experiment was performed to prove the mechanism of cellular uptake. For this purpose, MDA-MB -231, a CD44-overexpressing cancer cell line, was pretreated with free HA for 4 ​h before incubation with Epi-Nio-HA.

For confocal laser-scanning microscopy, the cells were seeded with 10^5^ ​cells density in 6-well glass-bottom dishes containing DMEM medium and 10% FBS and incubated for 24 ​h. Afterward, different groups were used to treat the cells. (Epi-Nio and Epi-Nio-HA). The incubated cells were rinsed with PBS after 2 ​h, and fixation was done using paraformaldehyde. Then, the cell nuclei were stained with Hoechst 33,342 for 5 ​min and washed with PBS. At last, the cells were observed by a confocal laser-scanning microscope (A1, Nikon, Switzerland) [30].

For the flow cytometry study, the chosen cells were seeded in 6-well plates (at a density of 5 ​× ​10^3^ ​cells/well) and incubated for 24 ​h. Then, the incubated cells were treated with different formulations as described above. After 2 ​h, the culture medium was removed and the cells were washed with PBS, trypsinized, and centrifuged (1200 ​rpm, 5 ​min). Finally, the cells were re-suspended in 500 ​μL PBS and evaluated by FACSCalibur flow cytometer. In addition, the control group was the cells without any treatment.

### 2.10 *In vivo* study

#### 2.10.1 *In vivo* study protocols and standards

In the current study, the *in vivo* experiments were performed strictly according to the protocols of the National Committee for Ethics in Biomedical Research. 20 female BALB/c inbred mice (weighing 18 ​± ​2 ​g, 6–8 weeks) were randomly divided into four groups (5 mice/group). Mice were kept under constant temperature (23 ​± ​2 ​°C), 55% humidity, and 12-h light-dark cycles.

#### 2.10.2 Breast cancer induction and treatment

4T1 cell line was used to induce breast cancer in mice. Briefly, after counting the cells, 2 million cells were suspended in sterile PBS 1X and then injected subcutaneously into the mice's flank. The measurement of tumor volume was performed with a digital caliper. When an initial tumor volume of 80 ​mm^3^ appeared, the treatment was started using free Epi, Epi-Nio, and Epi-Nio-HA formulations. The intravenous tail injection method was used to deliver the drug formulations on days 1, 7 and 14. After 21 days, the mice were sacrificed; then, the tumors were immediately excised and fixed in a 10% formalin buffer. Mice body weight were measured each three days during the treatment. The tumor volume measurements were firstly done at the beginning of treatment. After 21 days, the tumors were collected and their size has been measured at the end. The calculation of tumor volume was done by the below equation (Eq. (2)) [31];

$$\text{Volume ​}=\text{ ​}1\text{/}2\text{ ​× ​}\left({\text{width}}^{2}\right)\text{ ​× ​length}$$

#### 2.10.3 *In vivo* study design

The mice were separated into four experimental groups with 5 mice in each group:

Gr.1 - Cancer control.

Gr.2 - Mice treated with Epi at the dose of 5 ​mg ​kg.^−1^

Gr 3 - Mice treated with Epi-Nio at the dose of 2 ​mg ​kg.^−1^

Gr.4 - Mice treated with Epi-Nio-HA at the dose of 2 ​mg ​kg.^−1^

#### 2.10.4 Quantification of enzyme activity

After 21 days, the animals were euthanized and after opening the chest, blood samples were collected from the animals' hearts. Using the Abcam (Cambridge, MA, USA) kits, the activity levels of aspartate aminotransferase (AST), alkaline phosphatase (ALP), alanine aminotransferase (ALT), Creatinine, and blood urea nitrogen (BUN) in the samples were measured spectrometrically.

#### 2.10.5 Histopathology

Hematoxylin and eosin (H&E) staining were used for histopathological examination of the studied tissues. Tumors were classified histologically based on the Nottingham histological score system (Menten degree) [32]. Using this grading system, the number of nuclear characteristics, gland development, and mitotic activity were evaluated, which is presented in Table 1.

**Table 1:** Pathological scoring of pleomorphism, mitosis index, and invasion rate.

Score	Invasion	Pleomorphism	Mitosis
0	Absence of tumor cells in the dermis, and hypodermis	No	No
1	Penetration into dermis	Small, regular nuclei, and a shape	9–1
2	Infiltration into hypodermis	A moderate degree of difference in size, the shape of the nucleus, and hyperchromatic of the nucleus with the presence of nuclei	10–19
3	Penetration into subcutaneous muscle tissue	Severe degree of difference in nucleus size with hyperchromatic nuclei, and often with one or more nuclei identified	More than 20

### 2.11 Statistical analysis

All the values were reported as the mean ​± ​SD as a representative of at least three repetitions. Statistical analysis was carried out by Student's t-test and one-way analysis of variance (ANOVA). The graphs were plotted using GraphPad Prism 8 (GraphPAD Software Inc., La Jolla, CA, USA). The p-values lower than 0.05 were considered to have a significant difference.

## 3 Results

### 3.1 Optimization

The selected independent variables were lipid concentration, surfactant to cholesterol molar ratio, and surfactant type. The subsequent results of EE, size, and PDI were used for the optimization studies. Table 2 illustrates the results of optimization tests. According to Table 2, the size and PDI of Epi-Nio were found to be from 157.4 to 326.1 ​nm and 0.151 to 0.389, respectively. The EE% of Epi-Nio was from 62.82% to 83.23% which can be seen in Table 2. The ANOVA for the size, PDI, and EE are represented in Table S4. All the responses were fitted to the quadratic models and were polynomial (Table S5). The models were considered to be significant since their p values were less than <0.05. The statistical analysis indicates that the size and EE were considerably dependent on the independent factors (A, B, and C), whereas PDI was affected only by factor B (Surfactant/Cholesterol molar ratio). As seen in Table S6, closer R-squared values to adjusted R-squared values were anticipated. Indeed, if the adjusted R-squared value is closer to R-squared, the model can predict responses more precisely. The difference between R-square and adjusted R-squared values must be lower than 0.2 to be reliable. Furthermore, enough precision was employed to measure the signal/noise and to guarantee that this model could cover the design space. A ratio higher than 4 (the favorable value) was obtained for the size, PDI, and EE responses. The constant value of 1 ​mg/mL was considered as drug concentration in all formulations. Also, the surfactant/cholesterol ratio of 1.25, lipid concentration of 263.5 ​μmol, and span 60 surfactant were predicted to be the optimized formulation (Epi-Nio) in terms of EE, size, and PDI. The optimized responses acquired from the RSM method and related experimental data in the optimum conditions are shown in Table S7.

**Table 2:** Design of experiments using the RSM method to optimize the niosomal formulation of Epirubicin.

Run	Levels of independent variables					Dependent variables		
	Lipid (μmol)	Surfactant: Cholesterol (molar ratio)	Surfactant type	Cholesterol (mg)	Surfactant (mg)	Size (nm)	PDI	Entrapment Efficiency (EE) (%)
1	250	1	Span 60	48.33	53.83	157.4	0.197	77.96
2	200	1	Span 80	38.67	42.86	190.7	0.211	71.53
3	300	2	Span 80	38.28	86.15	271.2	0.335	82.1
4	200	1	Span 20	38.67	34.65	189.3	0.289	70.22
5	250	1	Span 80	48.33	53.58	175.9	0.208	78.24
6	250	2	Span 20	31.90	58.03	264.5	0.367	71.31
7	250	0.5	Span 80	64.76	35.36	267.5	0.289	74.53
8	250	0.5	Span 60	64.76	35.53	255.3	0.242	68.95
9	250	1	Span 80	48.33	53.58	186.3	0.229	75.41
10	300	0.5	Span 80	77.72	42.43	291.2	0.321	62.9
11	300	2	Span 20	38.28	69.64	282.9	0.321	75.36
12	300	1	Span 80	58.00	64.29	250.1	0.264	77
13	300	2	Span 60	38.28	86.55	284.1	0.334	80.35
14	200	1	Span 60	38.67	43.06	169.3	0.219	66.4
15	200	2	Span 80	25.52	57.43	228.3	0.259	71.53
16	200	0.5	Span 60	51.81	28.42	271.2	0.345	69.32
17	250	1	Span 20	48.33	43.31	205.8	0.189	69.59
18	250	1	Span 60	48.33	53.83	165.3	0.151	81.19
19	250	1	Span 20	48.33	43.31	201.4	0.229	67.18
20	300	1	Span 60	58.00	64.59	240.1	0.225	83.23
21	200	0.5	Span 20	51.81	22.87	288.9	0.315	62.82
22	250	2	Span 80	31.90	71.79	224.6	0.219	72.32
23	250	1	Span 80	48.33	53.58	198.3	0.258	73.27
24	300	0.5	Span 60	77.72	42.63	254.3	0.389	69.83
25	250	2	Span 60	31.90	72.13	223.7	0.185	73.89
26	250	0.5	Span 20	64.76	28.58	315.8	0.374	68.48
27	200	2	Span 20	25.52	46.43	227.1	0.23	72.03
28	250	1	Span 20	48.33	43.31	196.4	0.195	64.64
29	200	0.5	Span 80	51.81	28.29	301.2	0.328	72.32
30	250	1	Span 60	48.33	53.83	183.5	0.184	82.54
31	300	0.5	Span 20	77.72	34.30	326.1	0.365	67.32
32	200	2	Span 60	25.52	57.70	209.8	0.175	76.15
33	300	1	Span 20	58.00	51.97	231.7	0.286	71.85

**Fig. 2:**
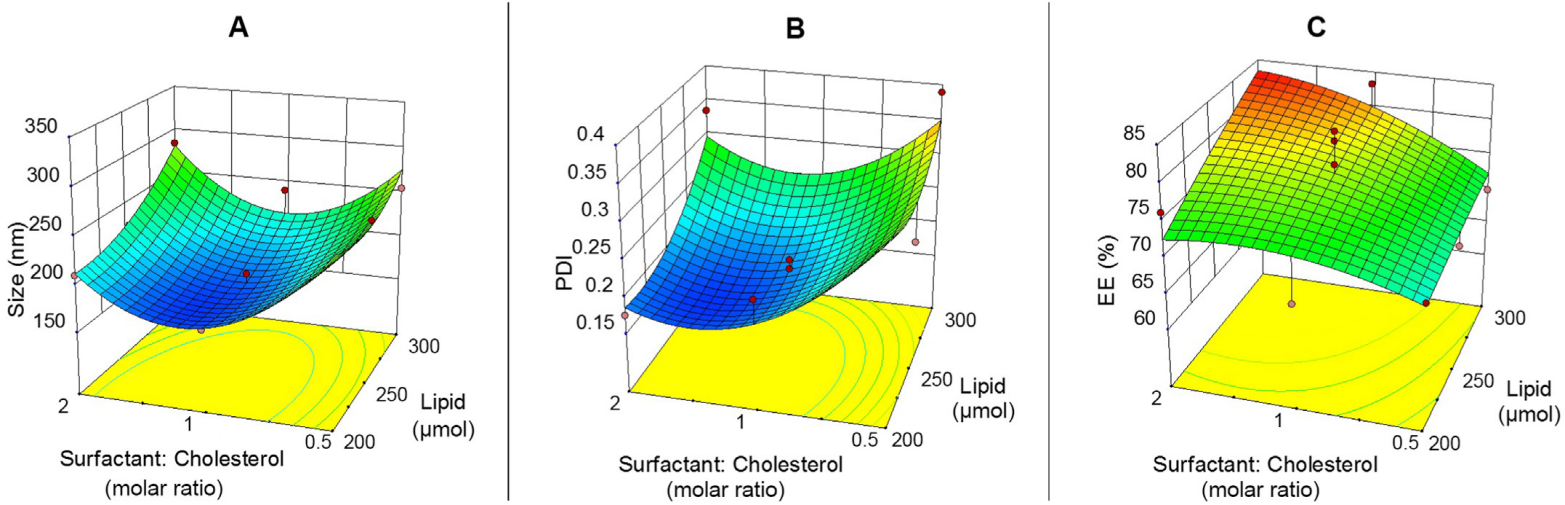
Central composite design method for A) size, B) PDI, and, C) EE as a function of the parameters (lipid concentration and surfactant/cholesterol ratio).

As it can be seen in Fig. 2, by increasing the surfactant/cholesterol ratio from 0.5 to 2, the size and PDI of niosomes at first decrease and then increase. However, this trend is different for EE which decreases by increasing the surfactant/cholesterol ratio. Besides, the size, PDI, and EE have risen by increasing the lipid concentration from 200 to 300 ​μmol.

### 3.2 DLS, FESEM, and TEM

**Fig. 3:**
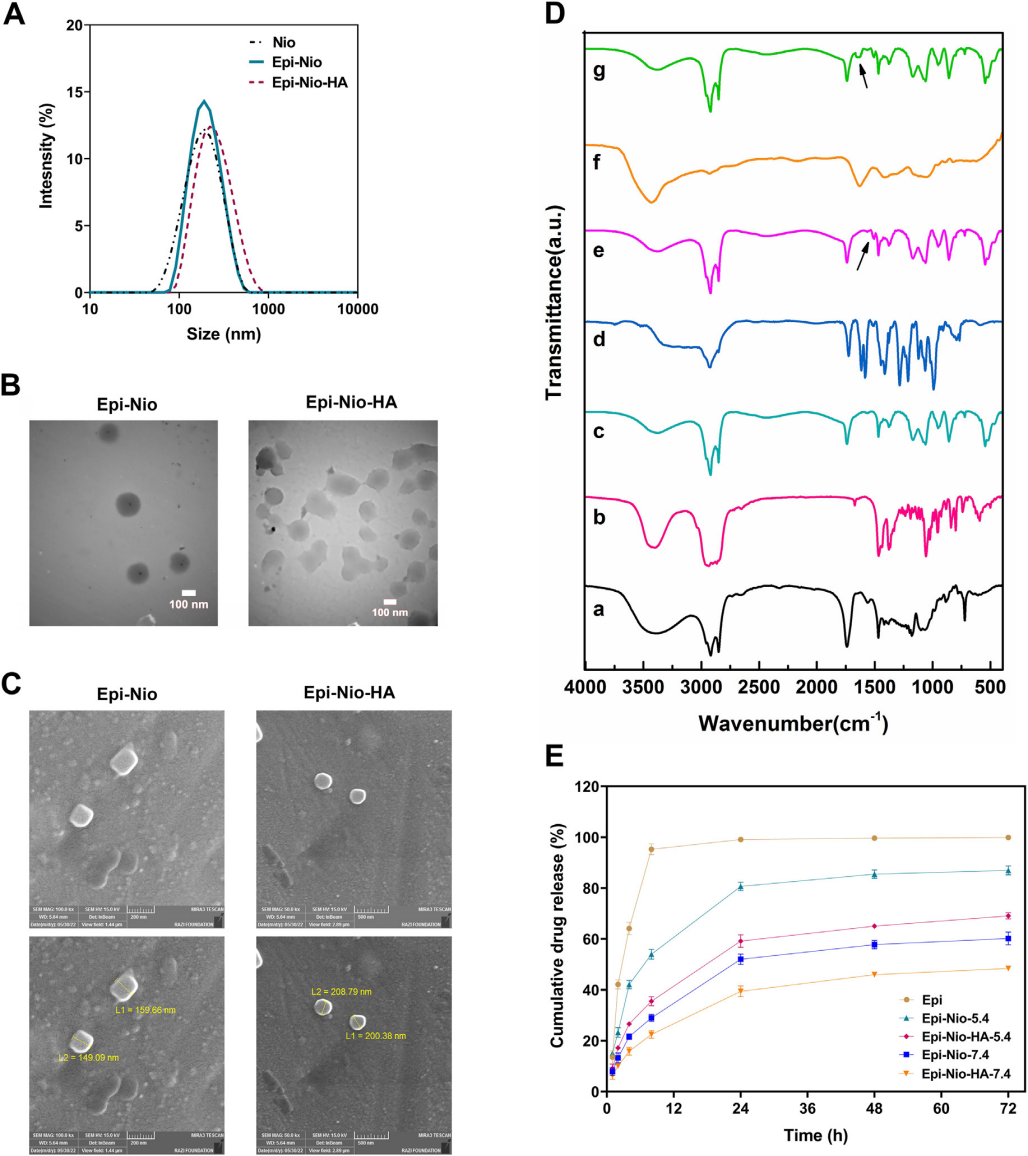
(A) Graphical presentation of the size distribution of Epi-Nio, and Epi-Nio-HA, Values represent mean ​± ​SD (n ​= ​3). (B) TEM image of the synthesized niosomal formulation with and without hydrogel coating (scale bar: 100 ​nm). (C) Field emission scanning electron microscopy (FESEM) images of Epi-Nio, and Epi-Nio-HA. (D) The FTIR spectra and peaks for every component of niosome, HA, and epirubicin: a) span60, b) Cholesterol, c) Nio, d) Epi, e) Epi-Nio, f) HA, g) Epi-Nio-HA (Arrows show amide groups of HA and C=C aromatic rings of epirubicin). (E) In vitro drug release pattern of epirubicin, Epi-Nio, and Epi-Nio-HA nanoparticles at pH 7.4 and pH 5.4 (n ​= ​3).

The size distribution of each group is displayed in Fig. 3A. The average size of bare niosomes (Nio), Epi-Nio, and Epi-Nio-HA groups were 167.2 ​nm, 186.5 ​nm, and 225.9 ​nm, respectively. In addition, PDI for Nio, Epi-Nio, and Epi-Nio-HA groups were 0.153, 0.145, and 0.160, respectively. The size and the morphology of dehydrated nanoparticles were analyzed using FESEM and TEM, which are shown in Fig. 3B and C. The FESEM results illustrated that the synthesized niosomes are spherical with a size range between 149.09 and 159.66 ​nm for Epi-Nio and a size range between 200.38 and 208.79 ​nm for Epi-Nio-HA. In addition, TEM results further prove the spherical morphology of nanoparticles and the maximum size of 180 ​nm and 270 ​nm for Epi-Nio and Epi-Nio-HA respectively. Besides, TEM results revealed uniform dispersity of nanoparticles and bilayer structure of niosomes. Moreover, as seen in FESEM and TEM images, the size of nanoparticles with HA coating was larger than bare niosomes.

### 3.3 Fourier-transform infrared (FTIR) spectroscopy

Fig. 3 D and Table S8 show the FT-IR spectra for each component of niosome, hydrogel, and epirubicin. Specifically, the niosome possesses specific peaks of C=O stretching (1746 ​cm^−1^), C–O stretching (1172 ​cm^−1^), aliphatic C–N stretching (1000–1250 ​cm^−1^), C–H stretching (2800–3000 ​cm^−1^), C–H symmetric stretching (1469 ​cm^−1^), and O–H stretching (3452 ​cm^−1^). The C–C stretching in aromatic ring (at 1506 ​cm^−1^) and C=C stretching (at 1674 ​cm^−1^) summits in cholesterol faded in the FT-IR spectra of niosomal formulation, confirming the cholesterol molecules entanglement in the lipid bi-layered membrane and noisome forming. The FT-IR spectrum for the Epi-Nio is similar to the spectrum of non-loaded niosomes but with slight shifts, corroborating the successful entrapment of epirubicin molecules in niosomal nanoparticles. Moreover, the major characteristic peaks of the epirubicin molecule have been disappeared in epirubicin-loaded nanoparticles and a shift for the C=C in the aromatic ring with the peak at 1505 ​cm^−1^ is observable, which further affirms the desirable epirubicin-encapsulation in the niosomes. Furthermore, the main characteristics peaks related to the Epi-Nio at 2800 ​cm^−1^ and 3000 ​cm^−1^ faded in the Epi-Nio-HA nanoparticles. Fig. 3D–g shows distinct peaks at 1635 ​cm^−1^ and 1503 ​cm^−1^ corresponding to the addition of hyaluronic acid to the Epi-Nio formulation and a shift for the C=C bond in the aromatic ring, respectively, which reveals successful functionalization of Epi-Nio with HA.

### 3.4 *In vitro* drug release and release kinetics study

*In vitro* drug release profiles of Epi-Nio and Epi-Nio-HA exhibited a two-phase pattern: a rapid release phase in the first 8 ​h (54% and 35% at pH 5.4; 29% and 22% at pH 7.4), followed by a very slow release phase till 72 ​h (Fig. 3E). As shown in Fig. 3E, in both Epi-Nio and Epi-Nio-HA groups, the release rate enhances at lower pH (5.4).

In order to examine the kinetic of epirubicin release from each formulation at a given pH, the obtained data were fitted to four kinetic models. The calculated regression coefficient values are summarized in Table 3. For each group, the model which shows the highest regression coefficient (R^2^) value (close to 1) is considered to be the best kinetic model for epirubicin release. As a result, epirubicin release without modification is described by first-order kinetic while the release from Epi-Nio and Epi-Nio-HA follows the Korsmeyer-Peppas model.

**Table 3:** Kinetic models and their calculated regression coefficient for Epirubicin release from the nanocarriers.

Model	Equation	Epi	Epi-Nio; 7.4	Epi-Nio; 5.4	Epi-Nio-HA; 7.4	Epi-Nio-HA; 5.4
Zero-Order	C_t_ ​= ​C_0_ – K_0_.t	R^2^ ​= ​0.4441	R^2^ ​= ​0.7965	R^2^ ​= ​0.7092	R^2^ ​= ​0.8297	R^2^ ​= ​0.7907
First-Order	Ln C_t_/C_0_ ​= ​– k_1_.t	R^2^ ​= ​0.8737	R^2^ ​= ​0.8485	R^2^ ​= ​0.8370	R^2^ ​= ​0.8686	R^2^ ​= ​0.8661
Korsmeyer–Peppas model	M_t_/M_∞_ ​= ​Kt^n^	R^2^ ​= ​0.7051 n ​= ​0.3842	R^2^ ​= ​0.9598 n ​= ​0.4755	R^2^ ​= ​0.9197 n ​= ​0.4062	R^2^ ​= ​0.9729 n ​= ​0.4822	R^2^ ​= ​0.9452 n ​= ​0.4556
Higuchi	C ​= ​K_H_√t	R^2^ ​= ​0.6149	R^2^ ​= ​0.9262	R^2^ ​= ​0.8646	R^2^ ​= ​0.9475	R^2^ ​= ​0.9228

### 3.5 Stability

**Fig. 4:**
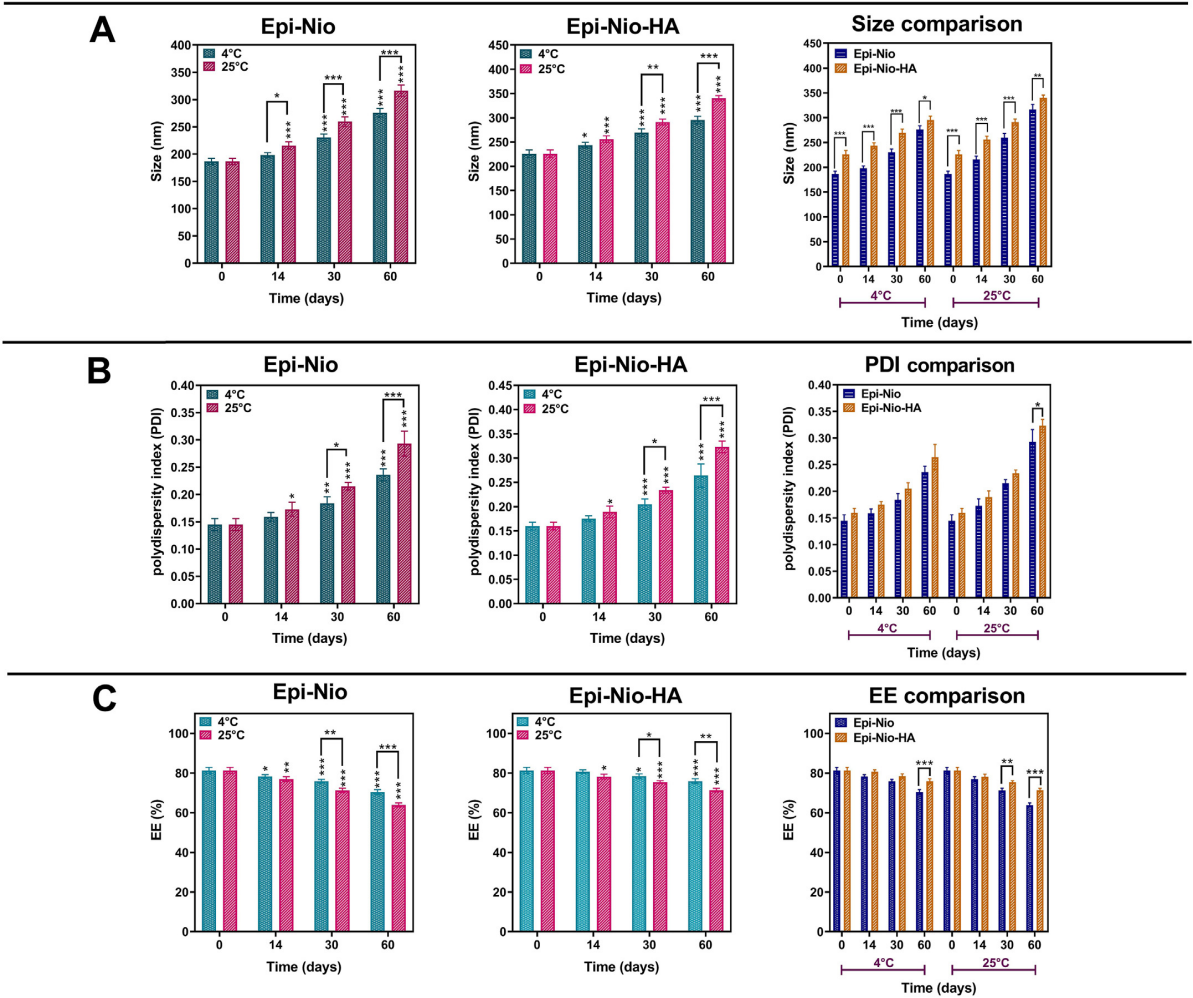
Stability of epirubicin loaded niosomes with and without HA coating, stored for 2 months at 4 ​± ​2 ​°C and 25 ​± ​2 ​°C. A) Sizes, B) PDI, and C) The EE% of the Epi-Nio and Epi-Nio-HA. (∗∗∗:p ​< ​0.001, ∗∗:p ​< ​0.01, ∗:p ​< ​0.05 using ANOVA test).

As can be seen in Fig. 4A and B, the size and PDI have an increasing trend with time and temperature. Thus, the stability of the optimized samples (optimization details in supplementary information) stored at 4 ​°C is higher than the samples at 25 ​°C. At different temperatures, the niosomes demonstrated an insignificant decrease in EE% during the first two weeks. However, major differences were observed in 30 days and 60 days for both groups (Fig. 4C) although the Epi-Nio-HA showed less reduction.

### 3.6 MTT, qRT-PCR, and flow cytometry

#### 3.6.1 Cytotoxicity effect of Epi, Epi-Nio, and Epi-Nio-HA

**Fig. 5:**
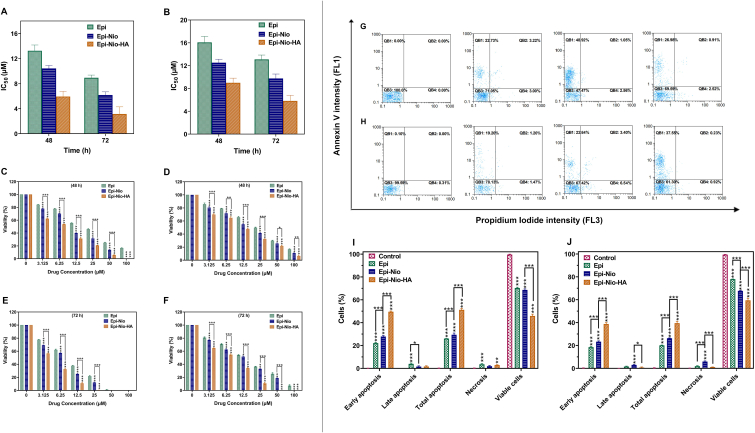
IC50 values for 4T1 (A) and SkBr3 (B) cells after 48 ​h and 72 ​h treatment. The *in vitro* cytotoxicity of 4T1 (C, E) and SkBr3 (D, F) cells which are illustrated after 48 ​h and 72 ​h of treatment with different concentrations (0, 3.125, 6.25, 12.5, 25, 50, 100 ​μM) of Epi, Epi-Nio, Epi-Nio-HA. Propidium Iodide vs Annexin V. QB1: early apoptotic cells, QB2: late apoptotic cells, QB3: viable cells, QB4: Necrotic cells, and total apoptosis for 4T1 (G, I) and SkBr3 (H, J) for 4T1 (G) and SkBr3 (H) cell lines (Control: Untreated sample). All data are presented as mean ​± ​SD (n ​= ​3 independent assays). (∗∗∗:p ​< ​0.001, ∗∗:p ​< ​0.01, ∗:p ​< ​0.05).

The cytotoxicity of Epi-Nio and Epi-Nio-HA against two cell lines (4T1 and SkBr3) was evaluated using MTT assay. The IC50 values of Epi-Nio have been significantly reduced in comparison with Epi, from 13.25 to 10.43 ​μmol ​mL^−1^ (4T1) and from 16.08 to 12.52 ​μmol ​mL^−1^ (SkBr3) after 48 ​h of incubation. Also, after 48 ​h of treatment, the IC50 values of Epi-Nio-HA against 4T1 and SkBr3 cells were 5.935 and 9.00 ​μmol ​mL^−1^, respectively (Fig. 5A and B). Free epirubicin (Epi) was considered as the control group. The treatment of 4T1 and SkBr3 cells with Epi-Nio-HA and Epi-Nio exhibited a higher inhibitory effect in comparison with epirubicin (Fig. 5C, D, E, and F), which is compatible with drug release results in acidic condition (Fig. 3E).

#### 3.6.2 Apoptosis assay (annexin V-FITC/PI)

To confirm the MTT assay results, the early and late apoptosis, as well as necrosis of breast cancer cells, were investigated using Annexin V-FITC/PI. Fig. 5 shows the apoptotic activity in 4T1 (Fig. 5G and I) and SkBr3 (Fig. 5H and J) cells using a flow cytometry apoptosis test. The results demonstrate that the administration of epirubicin-loaded niosomes with HA coating (Epi-Nio-HA) increased total apoptosis in both cancer cells (51.23% in 4T1 cells and 39.47% in SkBr3 cells) in comparison with the other two groups (Epi: 26.08% in 4T1 and 20.11% in SkBr3 cells; Epi-Nio: 29.42% in 4T1 and 26.34% in SkBr3 cells). Also, the necrosis was lower than apoptosis for all groups (Epi: 3.61% in 4T1 and 1.90% in SkBr3 cells; Epi-Nio: 1.93% in 4T1 and 5.93% in SkBr3 cells; Epi-Nio-HA: 2.89% in 4T1 cells and 1.09% in SkBr3 cells). In addition, the early apoptosis (i.e., 49.5% in 4T1 cells and 38.7% in SkBr3 cells) was remarkably higher when the cells were treated with Epi-Nio-HA formulation. The Epi-Nio-HA caused 27% higher cell death in 4T1 cells than that in SkBr3 cells.

#### 3.6.3 Real-time PCR

**Fig. 6:**
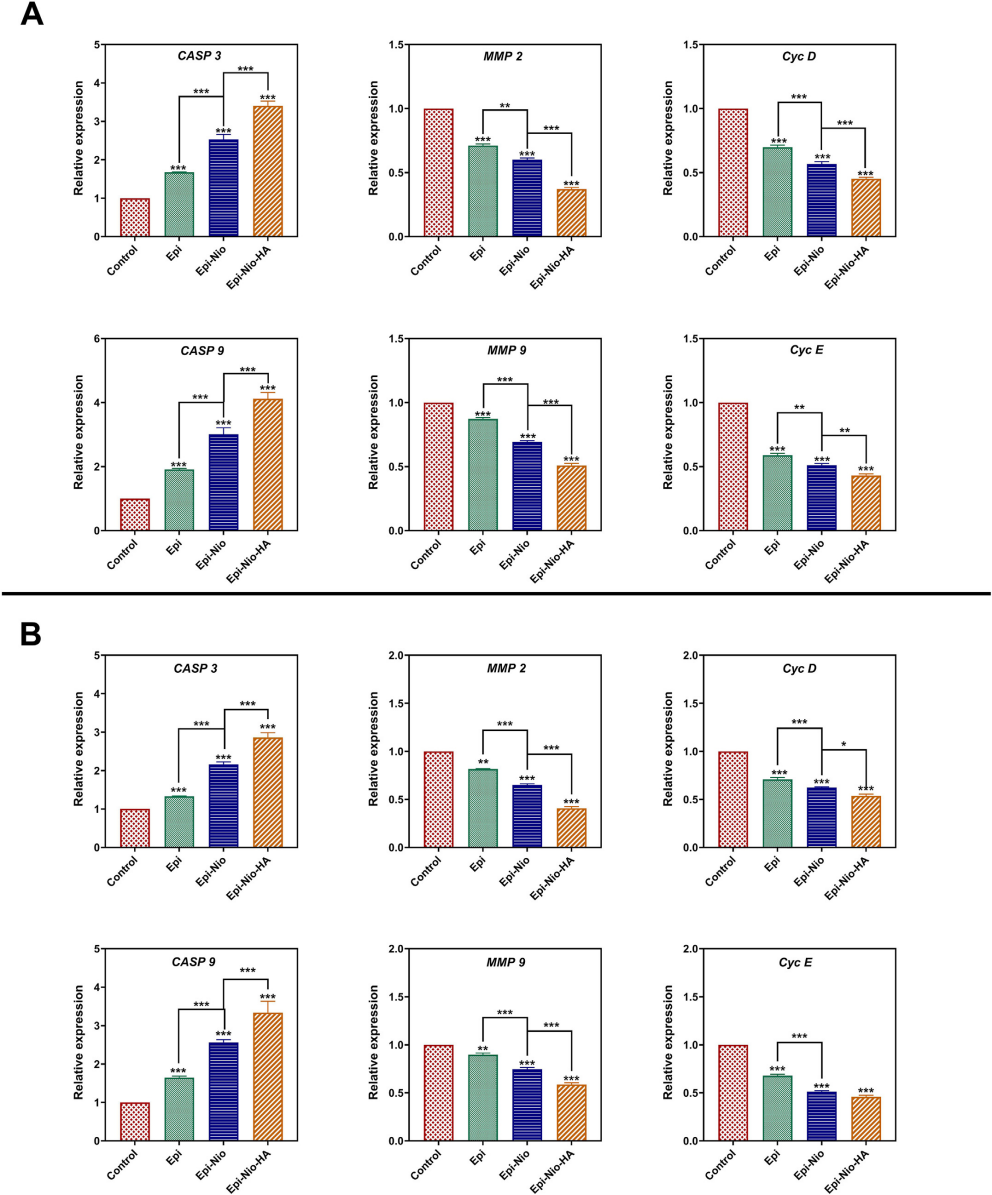
RT-qPCR analysis of cell cycle control, metastasis, and apoptotic pathways genes related to b-actin as housekeeping gene control after 72 ​h treatment of 4T1 (A), and SkBr3 (B) cell lines using Epi, Epi-Nio, Epi-Nio-HA. The statistical evaluation has been done according to ΔCT for Epi, Epi-Nio, Epi-Nio-HA compared to control as well as between adjacent groups (Epi-Nio, Epi-Nio-HA). Data are presented as mean ​± ​SD (n ​= ​3 independent assays). (∗∗∗:p ​< ​0.001, ∗∗:p ​< ​0.01, ∗:p ​< ​0.05).

To distinguish the efficiency of the synthetic nanoparticles (Epi-Nio and Epi-Nio-HA) for the inhibition of breast cancer, the expression of six different genes (i.e., CASP-3, CASP-9, and MMP-2, MMP-9, Cyc-D, and Cyc-E) involved in cell cycle, metastasis, and apoptotic pathways for 4T1 and SkBr3 breast cancer cell lines were assessed (Fig. 6A and B). The obtained data showed significant difference in gene expression level for the three groups (Epi, Epi-Nio, and Epi-Nio-HA) compared to the control group for both cell lines. In addition, the analysis confirmed statistical significance differences between Epi-Nio and Epi-Nio-HA for the expression of all genes in both cell lines, except for Cyclin E gene expression for SkBr3 cells. As shown in Fig. 6A and B, Epi-Nio-HA nanoparticles led to an increase in the extent of caspase-3 and caspase-9 expression; therefore, more apoptosis happened in comparison with Control and Epi-Nio groups for both cell lines. Interestingly, following administration of Epi-Nio-HA, MMP-2 and MMP-9 expressions were decreased. The synthesized Epi-loaded nanoparticles and the free drug could inhibit the cell cycle progression by downregulation of cyclin D and cyclin E expression.

### 3.7 Cellular uptake

To confirm the mechanism of cellular uptake and investigate the intracellular release behavior of the drug from nanoparticles, flow cytometry and confocal laser-scanning microscopy analyses were evaluated using different cell lines. To examine cell-specific targeted delivery of nanoparticles, MDA-MB-231 and MCF-7 ​cells were employed as CD44-overexpressing cancer cell lines, and NIH-3T3 cells were used as a CD44-negative cell line. Since epirubicin is a fluorescent drug, it was able to track the internalization and intracellular localization of nanoparticles. Moreover, the nuclei of the cells were stained by Hoechst 33,342 which emits blue fluorescence.

**Fig. 7:**
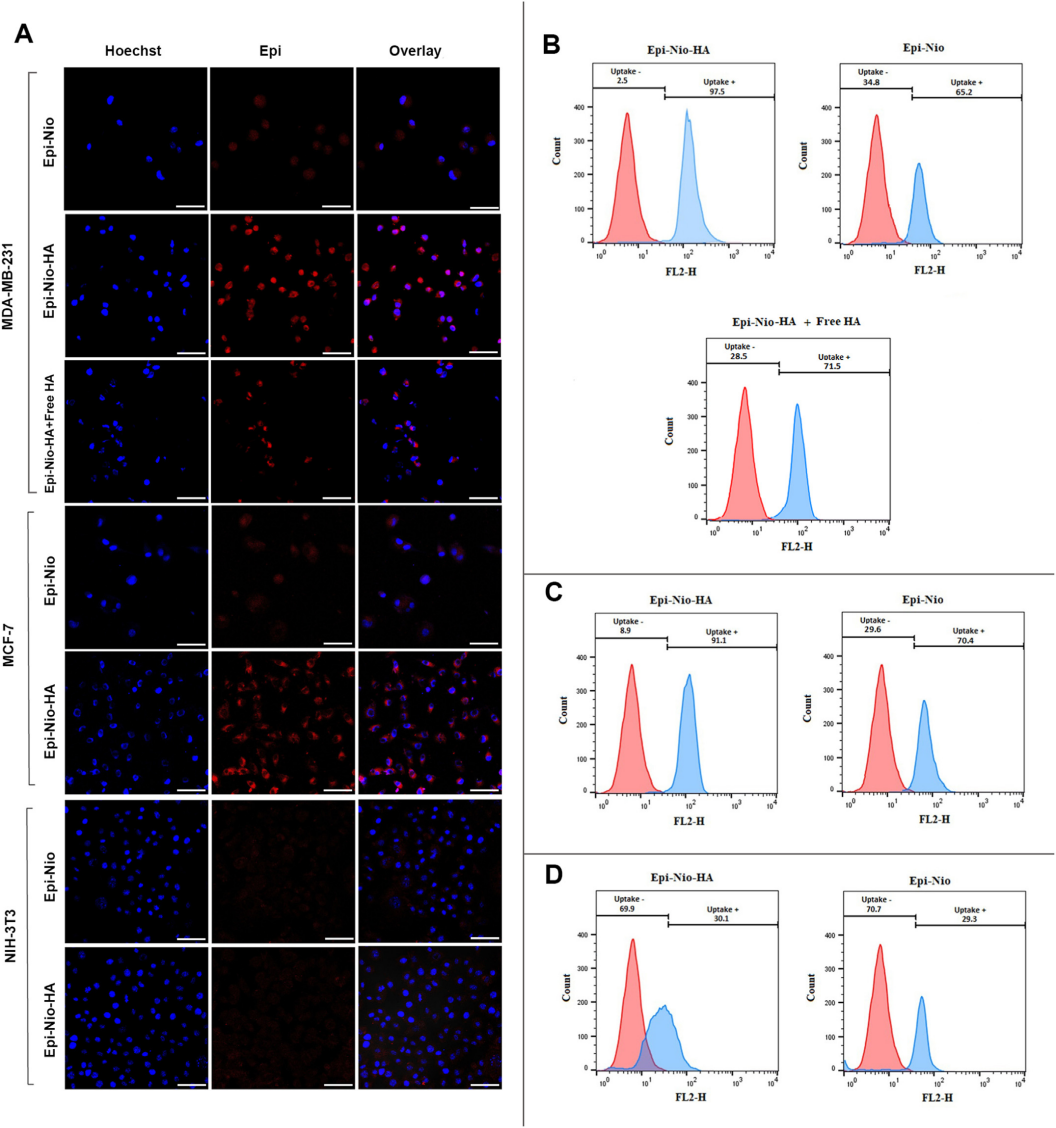
(A) Confocal laser-scanning microscopy images of different cell lines following 2 ​h incubation with Epi-Nio, Epi-Nio-HA, and Epi-Nio-HA ​+ ​free HA. Flow cytometry analysis of (B) MDA-MB-231, (C) MCF-7, and (D) NIH-3T3 cells following 2 ​h incubation. PBS was used as a control. The scale bar represents 50 ​μm.

Fig. 7A illustrates confocal microscopy images of three types of cell lines incubated with Epi-Nio and Epi-Nio-HA formulations for 2 ​h. The images obtained from the merged channels of Epi and Hoechst showed that Epi-loaded nanoparticles were internalized into the cells in 2 ​h, and the drug was released to reach cell nuclei. As shown in Fig. 7A, the nuclei were surrounded by the Epi-loaded nanoparticles internalized into the cells. Microscope images in Fig. 7A depicted that MDA-MB-231 and MCF-7 ​cells incubated with Epi-Nio-HA had a significantly stronger epirubicin fluorescence than those treated with Epi-Nio; thus, the HA-modified nanoparticles demonstrated higher cellular uptake than non-modified nanoparticles.

To verify the results of confocal microscopy analysis, the flow cytometry analysis was used to examine the cellular uptake of non-targeted (Epi-Nio) and targeted (Epi-Nio-HA) formulations in MDA-MB-231 (CD44-positive), MCF-7 (CD44-positive), and NIH-3T3 (CD44-negative) cell lines. As shown in Fig. 7B, **C**, and **D**, both formulations were taken up by three types of cells. The cellular uptake in CD44-positive MDA-MB-231 and MCF-7 ​cells enhanced for Epi-Nio-HA nanoparticles. The high fluorescence intensity was observed when our breast cancer cells were treated with Epi-Nio-HA compared to Epi-Nio nanoparticles (Fig. 7B and **C)**. The uptake of Epi-Nio-HA was 97.5% and 91.1% for MDA-MB-231 and MCF-7 ​cells, respectively. Moreover, 65.2% and 70.4% of internalization were detected for Epi-Nio in the MDA-MB-231 and MCF-7 ​cell lines, respectively. Moreover, the fluorescence intensity in MDA-MB-231 ​cells treated with Epi-Nio-HA nanoparticles decreased when the cells were preincubated with excess HA. The internalization of the Epi-Nio-HA formulation in MDA-MB-231 ​cells pretreated with excess HA was 71.5%, which was lower than that of Epi-Nio-HA (97.5%) in the same cells not pre incubated with free HA. On the other hand, for CD44-negative NIH-3T3 cells, the internalization of both formulations (non-targeted and targeted) was approximately the same, 29.3 and 30.1% for Epi-Nio and Epi-Nio-HA, respectively (Fig. 7D).

### 3.8 Histopathology

**Fig. 8:**
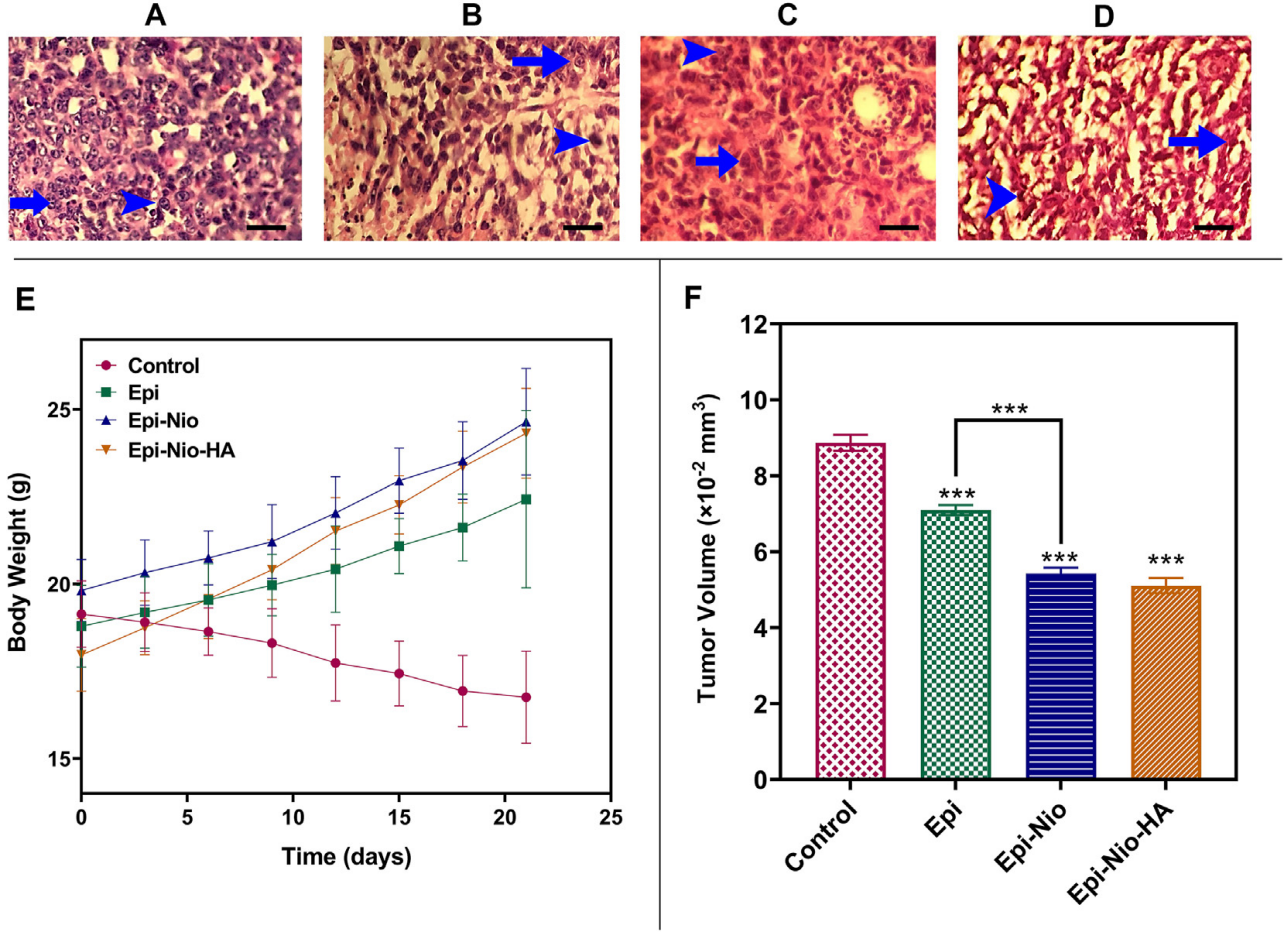
A–D: Microscopic imaging of mammary gland adenocarcinoma. H&E staining of cancer control (A), Epi (B), Epi-Nio (C), and Epi-Nio-HA (D); (Arrowhead: Cancer tissue and cell mitosis, Arrow: Polymorphism, original magnification: ×400, the scale bar represents 50 ​μm). The mice's body weight (g) (E); tumor volume ( ​× ​10^−2^ ​mm^3^) ​(F) of Epi, Epi-Nio, and Epi-Nio-HA treatment groups. (Data are reported as mean ​± ​SD and n ​= ​5; ∗∗∗:p ​< ​0.001, ∗∗:p ​< ​0.01, ∗:p ​< ​0.05).

The cancer control groups exhibited highly aggressive malignant breast tumor cells in the hypodermis, mitosis (Grade3), nuclear polymorphism (Grade3), and severe invasion (Grade3) (Table 4, Fig. 8A–D). In comparison to the control, in the Epi group, fewer tumor cells with mitosis and fewer cells with nuclei of pleomorphism were observed, but the invasion was still high. The Epi-Nio and Epi-Nio-HA groups were similar to the Epi-treated group in the study of nuclear pleomorphism. The Epi-Nio group showed some cells with mitosis, which was similar to the Epi group, but with a lower invasion. The number of cells with mitosis and the invasion was significantly reduced in the Epi-Nio-HA group in comparison with the control and other treatment groups.

**Table 4:** Pathological scoring of pleomorphism, mitosis index, and invasion rate in Cancer Control, Epi, and Epi-Nio-HA groups.

Group	Pleomorphism	Mitosis index	Invasion
Control	3	3	3
Epi	2	2	3
Epi-Nio	2	2	2
Epi-Nio-HA	2	1	1

### 3.9 Mice body weight and tumor volume

After the treatment period, the changes in mice's weights and tumor volumes were assessed. According to the results (Fig. 8E), the body weight of mice treated with Epi, Epi-Nio, and Epi-Nio-HA demonstrated a statistically significant increase in comparison with the control. However, no significant body weight changes were observed in Epi, Epi-Nio, and Epi-Nio-HA in comparison with each other (Fig. 8E). The changes in breast tumor volume between the control group and either Epi, Epi-Nio, or Epi-Nio-HA, were considerable, and a significant reduction was observed. In the comparison of Epi-Nio with Epi, and Epi-Nio-HA with Epi-Nio, a significant decrease was observed (Fig. 8F).

### 3.10 Renal and liver enzyme activity evaluation

Renal and liver serum biomarkers (BUN, Creatinine, AST, ALT, and ALP) were measured. The results demonstrated that the mice treated with Epi, Epi-Nio, and Epi-Nio-HA groups did not demonstrate any considerable changes in liver and renal enzymes activity compared to the healthy control (Table 5).

**Table 5:** Renal and liver enzymes activity assessment.

Enzyme	Healthy Control	Cancer Control	Epi	Epi-Nio	Epi-Nio-HA
BUN (mg/dL)	21.76 ​± ​2.02	35.43 ​± ​1.21	27.76 ​± ​1.42	25.14 ​± ​1.65	22.66 ​± ​2.14
Creatinine (mg/dL)	0.5 ​± ​0.1	1.67 ​± ​0.12	1.0 ​± ​0.21	0.7 ​± ​0.13	0.6 ​± ​0.24
AST (U/L)	105 ​± ​1.99	127.76 ​± ​2.55	122.76 ​± ​1.72	113.54 ​± ​2.76	103.86 ​± ​1.96
ALT (U/L)	115 ​± ​1.12	132.15 ​± ​3.99	121.56 ​± ​3.77	118.09 ​± ​3.65	117.55 ​± ​2.87
ALP (U/L)	267 ​± ​2.43	488.40 ​± ​3.66	311.43 ​± ​5.65	278.05 ​± ​4.51	234.78 ​± ​3.08

## 4 Discussion

This study aims at designing a novel therapeutic strategy using nanoparticles to target and treat breast cancer. To meet this goal, we have developed HA-decorated niosomal nanoparticles as a potential platform for targeted delivery of epirubicin to breast cancer cells, especially CD44-overexpressing cell lines. After optimizing the niosomal system, they were coated with HA to improve the targeting ability of nanoparticles.

As it is crystal clear, physicochemical properties such as smaller size, minimum PDI, and possessing maximum EE are the most effective factors on drug performance. The size and the EE of the niosomal formulation largely depend on surfactant type and the quantity of cholesterol (i.e., lipid) in niosomes [33].

In this study, we found span 60 a better surfactant in comparison to span 20 or span 80. This is also in agreement with previous studies [34]. The lowest phase transition temperatures of Span 20 and Span 80 are 16 ​°C and −12 ​°C, respectively, which keep them liquid at room temperature and prevent them from forming a gel at low cholesterol concentrations. On the contrary, Span 60, with a phase transition temperature of 53 ​°C, produced gel with or without cholesterol since it is solid at room temperature. Thus, it acts like a gelator in the system. Generally, span 60 is a suitable surfactant due to its high transition temperature and low hydrophilic-lipophilic balance (HLB) value (HLB ​= ​4.7); therefore, it forms niosomes of satisfactory properties [35]. Moreover, Span 80 is more hydrophobic (HLB ​= ​4.3) and it forms two phases of proniosomal liquids at low concentrations of cholesterol, and proniosomal gels of this condition are thermos-reversible [36]. Thus, although there is a consensus that the incorporation of cholesterol could improve the rigidity of the bilayer membrane [37], the effect of cholesterol content depends on the surfactant [38].

The results of DLS, FESEM, and TEM analyses in Fig. 3 clearly show that the loading of the drug in nanoparticles and coating of HA on niosomes increase the size of the nanoparticle. The niosomes' size obtained from microscopy analysis (FESEM and TEM) is smaller than that determined by DLS. This difference in size measurements could be due to different sample preparation in these methods and the process of drying before performing the microscopy imaging [39,40]. To clarify, the FESEM and TEM determine the accurate size of dehydrated nanoparticles which is equal to the core diameter. However, in the DLS method, the nanoparticles are in the hydrated state, and the determined size is larger because some molecules attach on the nanoparticle's surface. Thus, the hydrodynamic diameter obtained from the DLS method is much larger than the one determined by FESEM results [13,14].

According to Fig. 3E, *in vitro* drug release of our nano-formulations has shown a two-phase profile. The initial burst release might be due to epirubicin that is near the surface of nanoparticles which is released quickly. However, the sustained release phase could result from the presence of epirubicin molecules in the central core or the dense HA matrix [37]. The drug release rate has increased at a lower pH for both Epi-Nio and Epi-Nio-HA formulations. This might be attributed to the swelling and erosion of niosomes in acidic pH [41]. Moreover, the hydrolysis process of surfactants speeds up at lower pH, leading to the burst release of epirubicin in the acidic condition [42]. Additionally, the Epi-Nio-HA *in vitro* release results indicates that the cumulative release at pH 5.4 is 42% higher than that at pH 7.4; thus, the epirubicin release from the Epi-Nio-HA was pH-dependent. There will be more hydrogen bonds between hydrogel chains, resulting in the remaining the drug in the drug delivery system. However, since the rupture of the niosome dominated the system, the epirubicin at pH 5.4 was released rapidly, as compared with pH 7.4. Therefore, we concluded that Epi-Nio-HA could successfully and desirably control epirubicin release, which reduces side effects and improves therapeutic efficacy.

Considering R^2^ values, epirubicin release without any carrier follows the first-order kinetic model, indicating that the rate of drug release is proportional to the concentration of the remaining drug in the system. Nevertheless, epirubicin release from Epi-Nio and Epi-Nio-HA is described by the Korsmeyer-Peppas model. These results are compatible with recent studies on polymeric carriers [43]. The n values reveal that the non-Fickian diffusion mechanism (n ​> ​0.45) is dominant in epirubicin release from Epi-Nio-HA at two different pH values and from niosomes at pH 7.4. However, the n values of Epi-loaded niosomes at pH 5.4 (n ​< ​0.45) demonstrates that in acidic condition, the release is controlled by the Fickian diffusion mechanism [39,40,43,44].

According to Fig. 4, the stability of nanoparticles at 4 ​°C was more than 25 ​°C since the niosomes hydrophobic section is more rigid at a lower temperature. This can be attributed to their fusion or aggregation [45]. Surface energy could be size dependent, and the niosomes with smaller size have higher surface energy and tend to fuse to lower surface energy [46]. Another possible explanation for the increase in size is the swelling of the niosomes and hydrogel around them [47]. HA viscosity is lower at room temperature, and it thus provides the opportunity for more molecules to diffuse in the hydrogels and increase the system's volume [48]. Considering EE results, the high drug leakage at higher temperatures is probably due to the desorption from the niosomal surface and also because the lipid vesicles' fluidity increases at higher temperatures [49,50]. HA-coated niosomes have a highly viscous layer of hydrogel around them at 4 ​°C, which makes the drug diffusion slower and helps the system to keep the drug content for a prolonged delivery. Accordingly, the presence of hyaluronic acid in the formulation has a great effect on stability.

Previous studies have proved high biocompatibility and non-toxicity of bare niosomes and separate hyaluronic acid [12,17,37,51]. Moreover, hyaluronic acid can improve cell adhesion and interact with CD44 receptors on the surface of CD44-overexpressed cancer cells [51]. Therefore, CD44-mediated endocytosis can elucidate the reason for the higher cytotoxicity of Epi-Nio-HA in 4T1 and SkBr3 (Fig. 5). These findings are consistent with previous reports about hyaluronic acid conjugated nanoparticles [52]. The apoptosis results obtained from Annexin V-FITC/PI are compatible with cytotoxicity data acquired by the MTT assay. The higher cell death of 4T1 cells in comparison with SkBr3 cells could be due to overexpression of CD44 in the 4T1 cell line and therefore active targeting of hyaluronic acid [18,53].

Epirubicin has been found to have significant effects on the expression level of many genes within breast cancer cells [54]. Caspases are a kind of cysteine protease that can lead to apoptosis in cells via two different mechanisms (i.e., intrinsic pathways and extrinsic pathways) [55]. Caspase-3 activation is influenced by both intrinsic and extrinsic pathways, whereas caspase-9 activation is an intrinsic process. Since caspase-3 and caspase-9 can trigger rapid proteolysis of nuclear matrix proteins and DNA fragmentation, these genes are required in several processes related to the forming of apoptotic cells [56]. MMP-2 and MMP-9 are zymogens that are secreted primarily by tumor cells and stromal cells and play an essential role in extracellular matrix degradation, tumor invasion, and metastasis [57]. Cyclin E is a critical cyclin that modulates the phosphorylation of numerous proteins involved in cell proliferation, including NPAT [58], BRCA1 [59], and retinoblastoma protein [60]. Cyclin E overexpression has been observed in more than 25% of breast cancers, and it has recently been employed as an early predictive factor of the breast cancer initial stage [61]. Furthermore, the elevated cyclin D expression may influence DNA replication and repair, leading to genomic instability and increased gene amplification [62]. The qPCR analysis (Fig. 6) demonstrated that the synthesized nanoparticles desirably affected the gene expression in the studied breast cancer cell lines; resulting in down-regulation of the MMP-2, MMP-9, cyclin D, and cyclin E while up-regulation of caspase-3 and caspase-9. The obtained results are in good agreement with previous research on cancer drug delivery with HA coating for nanoparticles [,^63^]. Therefore, the findings indicated that Epi-Nio-HA nanoparticles have strong anticancer properties. The enhanced anticancer efficacy in the HA-functionalized group might be attributed to the improved uptake, mostly as a result of CD44-mediated endocytosis which highlights the need for evaluation of the uptake mechanism through confocal microscopy and flow cytometry in the following.

One of the most important features of breast cancer cells is their remarkable ability to migrate, leading to metastasis in the later stages, for which the solution is designing novel nanomedicines [64]. Recently, some studies have shown that chemotherapy-drug-loaded niosomes reduce cancer cell migration [13,65]. In this work, Epi-Nio-HA successfully reduced the migration of 4T1 and SkBr3 breast cancer cell lines by downregulating the expressions of MMP-2 and MMP-9 (Fig. S1). MMPs are enzymes that break down the extracellular matrix and basement membrane in the environment of cancer cells. MMP expression has been found to prevent tumor metastasis when it is reduced. Proteolytic enzymes MMP-2 and MMP-9 are overexpressed in cancer, resulting in increased tumor migration and invasion [66].

Interestingly, Hyaluronic acid has a strong affinity for the CD44 and hyaluronan-mediated motility (RHAMM) receptors, which are overexpressed by breast cancer cells, resulting in a strong targeted capacity for tumor cells [67]. Through receptor-mediated endocytosis, Hyaluronic based nanoparticles are thought to be an effective strategy for delivering anticancer medicines into CD44-overexpressing tumor cells, and subsequent enzymatic breakdown results in the nanoparticles content obtaining a sustainable release through the CD44-mediated endocytosis mechanism into the lysosome [[68], [69], [70], [71]].

According to the cellular uptake results (Fig. 7), it can be found that the CD44-positive cell lines can enhance the uptake of the Epi-Nio-HA nanoparticles approximately 3 times more than the CD44-negative cell lines (NIH-3T3). The enhanced cellular uptake of Epi-Nio-HA by MDA-MB-231 and MCF-7 breast cancer cells confirms the great efficiency of these nanocarriers as a successful formulation to target breast cancer cells. Furthermore, these results illustrate that HA-functionalized nanoparticles can enhance cellular uptake by approximately 1.5-fold in the cancer cells in comparison with non-targeted formulations that is an important achievement. In addition, cellular uptake of Epi-Nio-HA was decreased when an excess amount of free HA was used to pretreat MDA-MB-231 ​cells. As shown in Fig. 7, we detected the epirubicin fluorescence in the perinuclear region of cancer cells in Epi-Nio group and Epi-Nio-HA samples pretreated with free HA, while in the Epi-Nio-HA sample, the fluorescent drug was released into the cell nuclei.

More cellular uptake of Epi-Nio-HA nanoparticles in CD44-overexpressing cells could imply that Epi-Nio-HA nanoparticles were able to internalize into the cells using receptor-mediated endocytosis mechanism, via binding of HA to CD44 receptor. Interestingly, while MDA-MB-231 and MCF-7 ​cells both overexpress CD44, the fluorescence intensity of MDA-MB-231 ​cells treated with Epi-Nio-HA nanoparticles is more than MCF-7 ​cells incubated with the same formulation. This effect can be related to the higher expression level of CD44 at the surface of MDA-MB-231 ​cells in comparison with MCF-7 ​cells [[72], [73], [74]]. On the other hand, NIH-3T3 cells incubated with both targeted and non-targeted nanoparticles exhibited the same fluorescence signals, confirming the CD44-mediated endocytosis mechanism as NIH/3T3 cells are a CD44-negative cell line with a negligible expression level of CD44 receptors.

Flow cytometry results are in line with confocal microscopy results, supporting that the cellular uptake of Epi-Nio-HA nanoparticles is based on CD44-mediated endocytosis mechanism, which leads to a larger number of nanoparticles internalized into CD44-overexpressing tumor cells.

Recently, HA-coated nanoparticles for doxorubicin delivery have shown improved anticancer and tumor inhibition effects in mice bearing breast cancer tumors [53,]. In the present study, animals showed a decrease in tumor volume and an increase in body weight following treatment with Epi-Nio-HA (Fig. 8). It also presented its anticancer effects at the histopathological level by reducing mitotic cells, nuclear pleomorphism, and invasion. These results were consistent and complementary to our *in vitro* results. Thus, along with the cell-penetrating ability of niosomes, the modification with HA could enhance the *in vivo* targetableity, resulting in improvement of both *in vitro* and *in vivo* therapeutic efficacy. Moreover, the examination of enzymatic changes showed that Epi, Epi-Nio, and Epi-Nio-HA did not negatively affect the liver and renal organs.

## 5 Conclusion

In summary, this paper reports the development of an anticancer drug delivery system based on CD44-targeted nanoparticles for efficient epirubicin delivery to breast cancer cells. The synthesized Epi-Nio-HA nanoparticles were found to have a homogenous size on the nanoscale, revealed a sustained release, and remained stable in physiological condition. The Epi-Nio-HA group showed the most successful internalization into breast cancer cells, which was proved to be CD44-mediated. Our results confirmed that both Epi-Nio and Epi-Nio-HA groups improve epirubicin impact on breast cancer cells, including an increase in cytotoxicity and apoptosis, as well as inhibition of metastasis. Moreover, the *in vivo* results of Epi-Nio-HA nanoparticles illustrated the safe and efficient suppression of tumor growth in mice. Therefore, this formulated nanoparticle-based system can be considered as a promising methodology for developing safe and effective therapies against breast cancer.

## Credit author statement

A.M-K, I.A., E.M., and A.P.K. developed the idea and designed the experiments. A.M-K., S·K., H.M., A.M., M.A., and N.R. conducted the experiments. A.M-K, F.R.N., M.H., N.A., B·P., F.E.Y. and E.M. analyzed and interpreted the data. A.M-K., M.M., and E.M. lead the project. A.M-K., S·K., H.M., F.R.N., M.A., and N.R. wrote the manuscript. A.M-K., V.J., and E.M. revised the manuscript. E.M., M.M., and A.P.K edited the manuscript. Y.F. designed the schematic illustration. All authors have read the published version of the paper.

## Declaration of competing interest

The authors declare that they have no known competing financial interests or personal relationships that could have appeared to influence the work reported in this paper.
