# Supplementary material for: Engineered hyaluronic acid-decorated niosomal nanoparticles for controlled and targeted delivery of epirubicin to treat breast cancer
Source: Mater Today Bio. 2022 Jul 6;16:100349. doi: 10.1016/j.mtbio.2022.100349 (PMC9304880; doi:10.1016/j.mtbio.2022.100349)
Supplement: Multimedia component 1 [file mmc1.docx]

**Engineered Hyaluronic Acid-decorated Niosomal Nanoparticles for Controlled and Targeted Delivery of Epirubicin to Treat Breast Cancer**

Amirreza Mansoori-Kermani^a^, Sadaf Khalighi^a^, Iman Akbarzadeh^a^, Fazeleh Ranjbar Niavol^b^, Hamidreza Motasadizadeh^c^, Athar Mahdieh^c^, Vahid Jahed^d^, Masoud Abdinezhad^e^, Nikoo Rahbariasr^f,g^, Mahshid Hosseini^a^, Nima Ahmadkhani^a^, Behnam Panahi^a^, Yousef Fatahi^b,h^, Masoud Mozafari^i,^*, Alan Prem Kumar^j,k,^*, Ebrahim Mostafavi ^l,m,^*

^a^ Department of Chemical and Petrochemical Engineering, Sharif University of Technology, Tehran, Iran

^b^ Department of Stem Cells and Developmental Biology, Cell Science Research Center, Royan Institute for Stem Cell Biology and Technology, ACECR, Tehran, Iran

^c^ Department of Pharmaceutical Nanotechnology, Faculty of Pharmacy, Tehran University of Medical Sciences, Tehran, Iran

^d^ Rudolfs Cimdins Riga Biomaterials Innovations and Development Centre of RTU, Institute of General Chemical Engineering, Faculty of Materials Science and Applied Chemistry, Riga Technical University, Pulka St. 3/3, Riga, LV-1007, Latvia

^e^ School of Chemical Engineering, College of Engineering, University of Tehran, Tehran, Iran

^f^ Polymer Research Laboratory, Department of Chemistry, Sharif University of Technology, Tehran, Iran

^g^ Department of Chemistry, University of Victoria, Box 1700 STN CSC, Victoria, British Columbia V8W 2Y2, Canada

^h^ Nanotechnology Research Centre, Faculty of Pharmacy, Tehran University of Medical Sciences, Tehran, Iran

^i^ Department of Tissue Engineering and Regenerative Medicine, Faculty of Advanced Technologies in Medicine, Iran University of Medical Sciences, Tehran, Iran

^j^ Cancer Science Institute of Singapore and Department of Pharmacology, Yong Loo Lin School of Medicine, National University of Singapore, Singapore 117599, Singapore

^k^ NUS Centre for Cancer Research, Yong Loo Lin School of Medicine, National University of Singapore, Singapore

^l^ Stanford Cardiovascular Institute, Stanford University School of Medicine, Stanford, CA 94305, USA

^m^ Department of Medicine, Stanford University School of Medicine, Stanford, CA 94305, USA

*** Correspondence to:**

Ebrahim Mostafavi, PhD; Stanford Cardiovascular Institute, Stanford University School of Medicine, Stanford, California, USA; Email: [ebimsv@stanford.edu](mailto:ebimsv@stanford.edu); [ebi.mostafavi@gmail.com](mailto:ebi.mostafavi@gmail.com)

Masoud Mozafari, PhD; Currently at: Lunenfeld-Tanenbaum Research Institute, Mount Sinai Hospital, University of Toronto, Toronto, ON, Canada; Email: [mozafari.masoud@gmail.com](mailto:mozafari.masoud@gmail.com); [m.mozafari@utoronto.ca](mailto:m.mozafari@utoronto.ca)

Alan Prem Kumar, Cancer Science Institute of Singapore and Department of Pharmacology, Yong Loo Lin School of Medicine, National University of Singapore, Singapore 117599, Singapore; Email: [csiapk@nus.edu.sg](mailto:csiapk@nus.edu.sg)

**Supplementary material**

# Materials and Methods

# Optimization

**Table S1** and **S2** represent the levels of independent variables.

**Table S1**. Different levels for variables in the RSM optimization

| Level | -1 | 0 | +1 |
| --- | --- | --- | --- |
| A (Lipid, µmol) | 200 | 250 | 300 |
| B (Surfactant: Cholesterol, molar ratio) | 0.5 | 1 | 2 |

**Table S2**. Different levels for variables in the RSM optimization

| **Factor** | **Name** | **Type** | **Level1** | **Level2** | **Level3** |
| --- | --- | --- | --- | --- | --- |
| **C** | **Surfactant type** | **Categoric** | Span20 | Span60 | Span80 |

- 1. **Gene expression, Real-time PCR**

The data in **Table S3** represent the primer sequences of the target genes, CASP3, CASP9, MMP 2, MMP 9, Cyc D, Cyc E, and β-actin as a housekeeping gene.

**Table S3**. The sequence of primers used in PCR

| Gene | Sequence of Primers |
| --- | --- |
| *Cyclin D* | Forward: 5'- CAGATCATCCGCAAACACGC-3′  Revers: 5'- AAGTTGTTGGGGCTCCTCAG-3' |
| *Cyclin E* | Forward: 5'- CTCCAGGAAGAGGAAGGCAA-3′  Reverse: 5'- TTGGGTAAACCCGGTCATCA-3' |
| *Caspase 3* | Forward: 5'- CATACTCCACAGCACCTGGTTA-3′  Reverse: 5'- ACTCAAATTCTGTTGCCACCTT-3' |
| *Caspase 9* | Forward: 5’-CATATGATCGAGGACATCCAG-3  Reverse: 5’-TTAGTTCGCAGAAACGAAGC-3’ |
| *MMP 2* | Forward: 5'- TTGACGGTAAGGACGGACTC-3′  Reverse: 5'- CATACTTCACACGGACCACTTG-3' |
| *MMP 9* | Forward: 5'- GCACGACGTCTTCCAGTACC-3′  Reverse: 5'- CAGGATGTCATAGGTCACGTAGC-3′ |
| *β-actin* | Forward: 5-CATTGCTGACAGGATGCAGAAGG-3  Reverse: 5-TGCTGGAAGGTGGACAGTGAGG-3 |

# Scratch Assay

The 4T1 and SKBR3 cells migration was assessed via scratch assay. Both cell lines were cultured and seeded at 6*10^4^ cells per well in 24-well plates and incubated for 24 h at 37°C until they reached 70% confluency. Then, a scratch was created via a 200 μl pipette tip in each well. The floating cells have been gently washed with serum-free RPMI-1640 medium. After adding a fresh serum-free culture medium to each well, the cells were incubated with Epi, Epi-Nio, and Epi-Nio-HA for 72 hr. Then, the cells were rinsed with sterile PBS and finally photographed with an inverted microscope and analyzed with ImageJ software (NIH, USA).

# Results and discussion

- 1. **Optimization**

The optimized values obtained from the RSM method and related experimental data at the optimum conditions are shown in **Table S7**.

**Table S4**. Analysis of variance for the quadratic polynomial model for Size, PDI, and EE

| **Source** | **f-Value** | **p-value**  **prob > f** |  |
| --- | --- | --- | --- |
| **Size (nm)** | | | |
| **Model** | 25.38 | < 0.0001 | Significant |
| **A** | 30.56 | < 0.0001 |  |
| **B** | 30.46 | < 0.0001 |  |
| **C** | 9.87 | 0.0009 |  |
| **AB** | 9.58 | 0.0055 |  |
| **A^2** | 11.06 | 0.0032 |  |
| **B^2** | 139.48 | < 0.0001 |  |
| **PDI** | | | |
| **Model** | 5.77 | 0.0003 | Significant |
| **A** | 7.18 | 0.0140 |  |
| **B** | 9.62 | 0.0054 |  |
| **C** | 3.53 | 0.0476 |  |
| **A^2** | 6.15 | 0.0217 |  |
| **B^2** | 20.31 | 0.0002 |  |
| **EE (%)** | | | |
| **Model** | 3.28 | 0.0094 | Significant |
| **A** | 4.64 | 0.0429 |  |
| **B** | 11.25 | 0.0030 |  |
| **C** | 6.84 | 0.0052 |  |

**Table S5**. Predicted models of Epi loaded niosomes

| **Models** |
| --- |
| **Size (Span20)** = +202.74+22.57 * A- 26.05* B +13.56* A * B + 18.30 * A2+ 65.00 * B2  **Size (Span60)** = +174.01+21.37 * A- 10.53* B +13.56* A * B + 18.30 * A2+ 65.00 * B2  **Size (Span80)** = +189.59+15.38 * A- 22.63* B +13.56* A * B + 18.30 * A2+ 65.00 * B2 |
| **PDI (Span20)** = +0.230 + 0.023 * A- 0.024* B + 0.020* A * B +0.037 * A2+ 0.067 * B2  **PDI (Span60)** = +0.183 + 0.035 * A- 0.047* B + 0.020* A * B +0.037 * A2+ 0.067 * B2  **PDI (Span80)** = +0.208 + 0.020 * A- 0.021* B + 0.020* A * B +0.037 * A2+ 0.067 * B2 |
| **EE (Span20)** = +70.55+ 1.58 * A + 3.35* B +1.88* A * B - 0.37 * A2- 2.17 * B2  **EE (Span60)** = +76.82+ 3.55 * A + 3.72* B +1.88* A * B - 0.37 * A2- 2.17 * B2  **EE (Span80)** = +75.13+ 1.10 * A + 2.70* B +1.88* A * B - 0.37 * A2- 2.17 * B2 |

**Table S6**. Results of regression analysis for responses (size, PDI, and EE)

| **Response** | **R-squared** | **Adj R- Squared** | **Adeq Precision** | **Lack of fit** |
| --- | --- | --- | --- | --- |
| **Particle size** | 0.9300 | 0.8934 | 16.409 | 0.1265 |
| **PDI** | 0.7514 | 0.6212 | 7.115 | 0.0507 |
| **EE (%)** | 0.6318 | 0.4390 | 7.697 | 0.0622 |

**Table S7**. The optimized values obtained by RSM method and the experimental data for the same values under the optimum conditions

| **Parameter** | **Predicted by RSM** | **Experimental Data**  **(Epi-Nio)** | **Epi-Nio-HA** | **Niosome**  **(Nio)** |
| --- | --- | --- | --- | --- |
| **Average size (nm)** | 183.758 | 186.50±5.75 | 225.90±7.94 | 167.20±6.90 |
| **PDI** | 0.189 | 0.145±0.011 | 0.160±0.008 | 0.153±0.005 |
| **Entrapment Efficiency (EE) (%)** | 78.720 | 81.29±1.57 | 81.29±1.57 | - |

- 1. **Fourier-Transform Infrared (FTIR) Spectroscopy**

**Table S8** shows the FT-IR spectra and peaks for every component of niosome, hydrogel, and epirubicin.

Table S8. The main characteristic peaks for FT-IR spectra of different samples/chemicals

| **Sample, chemicals** | **Peak cm^-1^** | **Description** |
| --- | --- | --- |
| **Epirubicin** | 2918 | C–H stretching |
|  | 1720 | C = O stretching |
|  | 1400-1600 | C=C aromatic rings |
| **Span 60** | 1125 | C–O stretching |
|  | 2800-3000 | C-H stretching |
|  | 3452 | OH stretching |
| **Cholesterol** | 1747 | C = O stretching |
|  | 2800-3000 | C-H stretching |
|  | 3452 | OH stretching |
|  | 1035-1378 | CH_2_ bending and CH_2_ deformation |
|  | 1506 | C-C stretching in aromatic ring |
|  | 1674 | C=C stretching |
| **Niosome** | 1125 | C–O stretching |
|  | 1747 | C = O stretching |
|  | 2800-3000 | C-H stretching |
|  | 3452 | OH stretching |
|  |  |  |
| **Noisome- Epirubicin** | 1505 | C=C aromatic rings |
| **Noisome-Epirubicin- HA** | 1635 | amide groups OF HA |
|  | 1503 | C=C aromatic rings OF Epirubicin |
| **Hyaluronic Acid (HA)** | 1659 | Amide group |
|  | 1436 | Carboxyl group stretching |

# Scratch (Cell Migration) Assay

The scratch test was performed to evaluate the effects of Epi, Epi-Nio, and Epi-Nio-HA on migration of 4T1 (**Fig. S1A**), and SKBR3 (**Fig. S1B**) cells. As shown in (**Fig. S1C**), the scratch width (μm) in 4T1 cells that were treated with Epi, Epi-Nio, and Epi-Nio-HA were 37.245±3.095, 68.91±2.76, and 85.73±3.08 μm respectively. The scratch width in these groups increased significantly compared to the control. Also, in comparing the scratch width of the Epi-Nio with Epi, and Epi-Nio-HA with Epi-Nio, a statistically significant increase was observed. In (**Fig. S1D**), which shows the effects of Epi, Epi-Nio, and Epi-Nio-HA on the migration of SKBR3 cells, the scratch width was 26.095±2.77, 55.08±1.79, and 72.385±1.73 respectively, indicating a significant increase compared to the control. Additionally, comparing the scratch width of the Epi-Nio with Epi and Epi-Nio-HA with Epi-Nio, a statistically significant increase was observed.


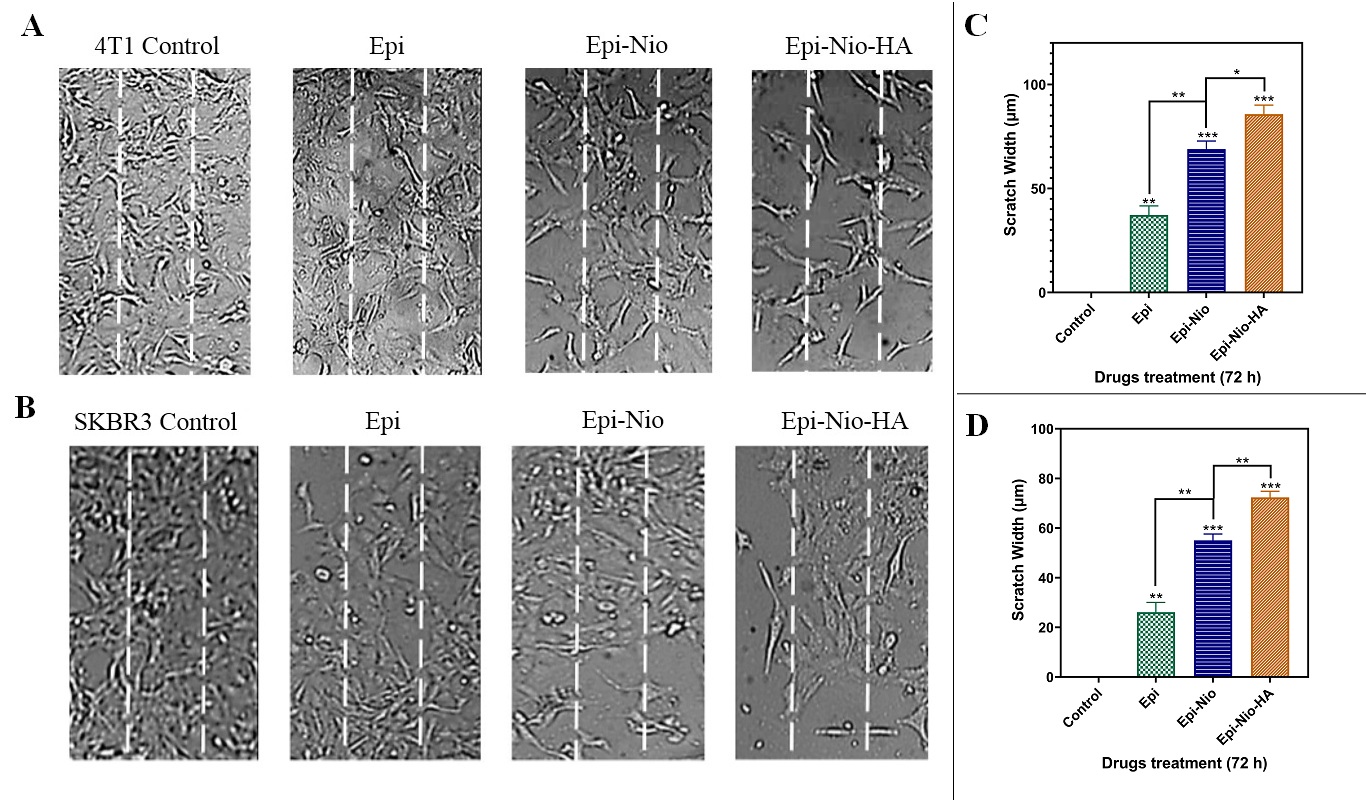


**Fig. S1.** Detection of 4T1, and SKBR3 cancer cells migration using a scratch assay. Microscopic images of 4T1 (A), and SKBR3 cells (B); the inhibitory effects of Epi, Epi-Nio, and Epi-Nio-HA on the migration of the 4T1 (C), and SKBR3 (D) breast cancer cells after 72h of treatment; Data are represented as mean ± SD and n=3; p<.001***, p<0.01 **, p<0.05*.
